# Human viruses: discovery and emergence

**DOI:** 10.1098/rstb.2011.0354

**Published:** 2012-10-19

**Authors:** Mark Woolhouse, Fiona Scott, Zoe Hudson, Richard Howey, Margo Chase-Topping

**Affiliations:** Centre for Immunity, Infection and Evolution, University of Edinburgh, Ashworth Laboratories, Kings Buildings, West Mains Road, Edinburgh EH9 3JT, UK

**Keywords:** discovery curves, emerging infectious diseases, public health, risk factors, surveillance

## Abstract

There are 219 virus species that are known to be able to infect humans. The first of these to be discovered was yellow fever virus in 1901, and three to four new species are still being found every year. Extrapolation of the discovery curve suggests that there is still a substantial pool of undiscovered human virus species, although an apparent slow-down in the rate of discovery of species from different families may indicate bounds to the potential range of diversity. More than two-thirds of human viruses can also infect non-human hosts, mainly mammals, and sometimes birds. Many specialist human viruses also have mammalian or avian origins. Indeed, a substantial proportion of mammalian viruses may be capable of crossing the species barrier into humans, although only around half of these are capable of being transmitted by humans and around half again of transmitting well enough to cause major outbreaks. A few possible predictors of species jumps can be identified, including the use of phylogenetically conserved cell receptors. It seems almost inevitable that new human viruses will continue to emerge, mainly from other mammals and birds, for the foreseeable future. For this reason, an effective global surveillance system for novel viruses is needed.

## 1. Introduction

Following on from the discovery of tobacco mosaic virus in 1892 and foot-and-mouth disease virus in 1898, the first ‘filterable agent’ to be discovered in humans was yellow fever virus in 1901 [1]. New species of human virus are still being identified, at a rate of three or four per year (see below), and viruses make up over two-thirds of all new human pathogens [2], a highly significant over-representation given that most human pathogen species are bacteria, fungi or helminths. These new viruses differ wildly in their importance, ranging from the rare and mild illness due to Menangle virus to the devastating public health impact of HIV-1.

In this paper, we take an ecological approach to studying the diversity of human viruses (defined as viruses for which there is evidence of natural infection of humans). First, we describe and analyse temporal, geographical and taxonomic patterns in the discovery of human viruses (§2). We then consider the processes by which new human viruses emerge (§3). There are a number of definitions of ‘emergence’ [3]; here, we are interested in all stages of the process by which a virus shifts from not infecting humans at all to becoming a major human pathogen. As experiences with HIV-1 and new variants of influenza A (and also with novel animal pathogens such as canine parvovirus [4]) show, this shift can occur rapidly, over time scales of decades, years or even months.

Of course, not all newly identified human virus species are ‘new’ in the sense that they have only recently started to infect humans; many of them have been present in humans for a considerable time but have only recently been recognized (see [2] for a more detailed discussion). Moreover, we recognize that ‘species’ itself is an imprecise designation, especially for viruses such as influenza A where different serotypes can have very different epidemiologies and health impacts. Indeed, the demarcation between genus, species complex, species and serotype (or other designations of sub-specific variation) can be somewhat arbitrary. Nonetheless, a study of currently recognized ‘species’ is a natural starting point for attempts to characterize and interpret patterns of virus diversity.

## 2. Virus diversity and discovery

### (a) Survey of human viruses

As a starting point for our survey, we used a previously published database (see [5]) obtained by systematically searching the primary scientific literature up to and including 2005 for reports of human infection with recognized virus species, using species as defined by the International Committee on Taxonomy of Viruses (ICTV) [6]. The list of viruses was updated if either a new species that can infect humans had been described in the literature and also recognized by the ICTV, if a known species had been found in humans for the first time, or if there had been a change in species classifications by the ICTV (notably for the human papillomaviruses and the vesicular stomatitis viruses).

The year of discovery was taken to be the year of publication of the first report of human infection. The place of discovery was determined from the original report and recorded as the location of the diagnostic laboratory or, in the few instances where this was not clear, the address of the first author of the report. We did not attempt to locate the case itself, as this information was often lacking.

We obtained a list of 219 ICTV-recognized virus species that have been reported to infect humans. 23 virus families were represented by species in this list.

### (b) Discovery curve

**Figure 1.**
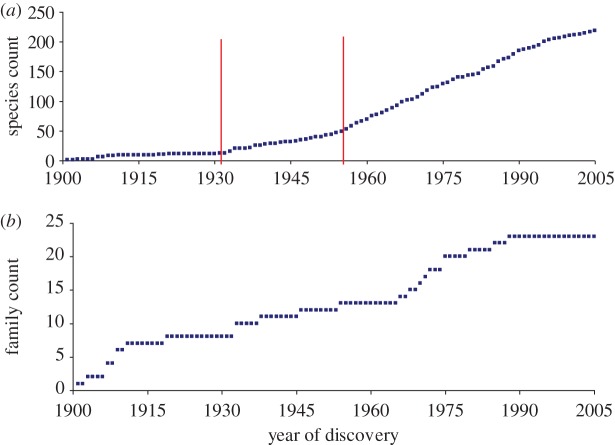
Discovery curves for human viruses. (*a*) Virus discovery curve by species. Cumulative number of species reported to infect humans. Statistically significant upward breakpoints are shown (vertical lines). (*b*) Virus discovery curve by family. Cumulative number of families containing species reported to infect humans.

The discovery curve is an ecological tool for estimating species diversity [7] comprising a simple plot of the cumulative number of species against time or sampling effort. Discovery curves are normally drawn for defined geographical areas; here we equate ‘humans’ with a delimited habitat for viruses. The discovery curve for human virus species is shown in figure 1*a*.

As with all discovery curves, our curve reflects a number of different factors, including: (i) the technology available for detecting viruses (table 1); (ii) the effort invested in detecting new viruses; (iii) the ‘visibility’ of different virus species, e.g. as a function of how common they are and the nature of any disease caused; (iv) virus taxonomy and the rules for designating a ‘species’; (v) the emergence of new virus species that did not previously infect humans.

**Table 1.** Major developments in the technology of virus discovery (adapted from [8]).

year	technology
1890s	filtration
1929	complement fixation
1948	tissue culture
1970s	monoclonal antibodies
1985	polymerase chain reaction (PCR)
2000s	high throughput sequencing

Piecewise linear regression revealed two statistically significant (*p* < 0.05) upswings in the rate of virus discovery: in 1930 (95% confidence intervals (CIs) 1927–1933) and in 1954 (1952–1955). Since 1954 the mean rate of discovery has been 3.37 species per year with variance 3.35, consistent with a Poisson process. However, there has been a slight but statistically significant downward trend in the rate of discovery (a linear regression of (count per year)^0.5^ against year has slope −0.010, 95% CIs −0.020 to 0.0, *p* = 0.049).

### (c) Geography and taxonomy

**Figure 2.**
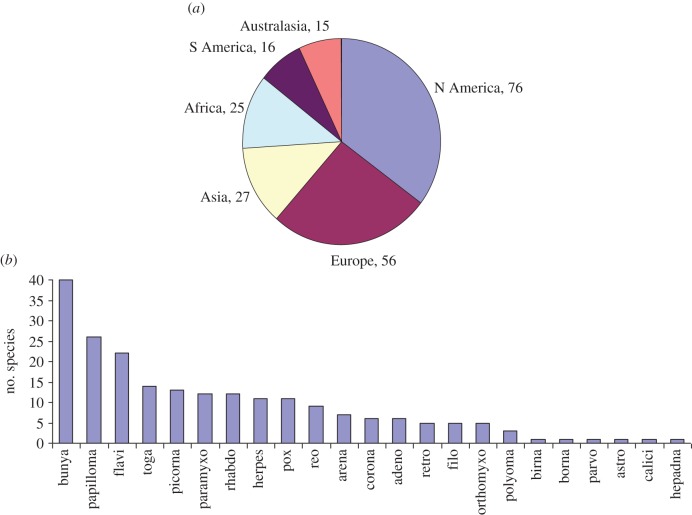
Patterns in human virus diversity. (*a*) A pie chart showing the continent where human virus species were first reported (*n* = 215, with four species not assigned to a continent). (*b*) Species abundance histogram for human viruses by family. Twenty three families are represented; six virus species remain unassigned to a family.

Numbers of species discovered by continent are shown in figure 2*a* (ignoring four species for which the location of discovery could not be determined). That over 60 per cent of species were first discovered in North America or Europe almost certainly reflects considerable ascertainment bias [9,10]. Rates of discovery by continent have, perhaps unsurprisingly, been very variable through time but with no clear patterns; the only notable trend in the last 15 years has been a higher rate of discovery in Australasia.

Numbers of species by family are shown in figure 2*b*. The family containing the most human virus species is the Bunyaviridae with 40; six families contain just one human virus species. These numbers are too small for statistical analysis of rates of discovery: the most notable trend is that only a single new pox virus has been discovered since 1972 (compared with 10 up to that date). Nor are there any striking patterns using other classifications such as RNA viruses versus DNA viruses.

### (d) Projecting the discovery curve

Following the approach described previously [5], we modelled human virus discovery since 1954, assuming a total number of species available to be discovered—the species pool—of *N* virus species, each discovered in any given year with probability *p*. We considered fitting a distribution for values of *p*; however, provided that the individual *p* values are low, there was minimal improvement in model fit. The model was fitted to the data and evaluated using Markov chain Monte Carlo (MCMC) methods with flat prior information to calculate profile likelihood confidence intervals and the best fit parameters. The model defines the expected number of discovered viruses in year *t*, *λ_t_*, as binomially distributed so that2.1
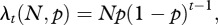
where year *t* = 1 corresponds to 1954.

However, the binomial distribution B(*N*, *p*) can be accurately approximated by a Poisson distribution with parameter *Np* for the range of values of *N* and *p* of interest. Thus, for a set of model parameters, the likelihood of observing data *X* = {*x_i_*}, the number of viruses discovered over years 1 to *k*, is given by2.2
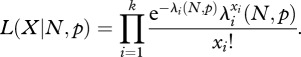


We compared the model with the observed data by calculating the mean, trend in the mean and variance for the number of virus species discovered per year (based on 5 million simulations using best fit parameter values). The model reproduces the observed data well: observed mean and variance 3.37 and 3.35, respectively; fitted mean and variance 3.36 and 3.41, respectively. Parameter estimates, however, are very uncertain owing to an unavoidable strong correlation between *N* and *p* [5]. The estimate of *N* is of particular interest: this has a central value of 484 (i.e. 265 species remaining to be discovered), a lower 95% CI of 308 (89 remaining), an upper 90% CI > 2000 and an upper 95% CI that is undefined. Thus, although there is considerable uncertainty as to the size of the human virus species pool, this analysis suggests that there are at least dozens of new species to be discovered, and possibly a very much larger number.

To make shorter term projections, the model was extrapolated to year 2020, calculating 95% posterior prediction intervals using 2 million model simulations, taking into account parameter uncertainty and model stochasticity. An upper limit for *N* was set at the 90% upper confidence interval. This gave a projected number of new virus species of 36 (95% CIs 20–57), corresponding to an average 2.4 species per year. This projection, of course, makes no allowance for any improvements in virus detection technology nor changes in discovery effort.

### (e) Recently discovered viruses

From our systematic literature review, we identified at least 14 putative new species of human virus first reported during the 5 years 2005 to 2009 inclusive (table 2), though this list is almost certainly incomplete. Clearly (subject to recognition of these new viruses as distinct ‘species’ by the ICTV), the projection described in §2*d* looks likely to be met. Indeed, it would be unsurprising if it were exceeded, given the considerable recent interest in virus discovery and the advent of high throughput sequencing as a detection tool.

**Table 2.** Examples of putative new human virus species reported from 2005 to 2009 [11–24].

virus name	family
human bocavirus	Parvoviridae
parvovirus 4	Parvoviridae
KI polyomavirus	Polyomaviridae
Melaka virus	Reoviridae
WU polyomavirus	Polyomaviridae
astrovirus MLB1	Astroviridae
Bundibugyo ebolavirus	Filoviridae
human bocavirus 2	Parvoviridae
human cosaviruses A-D	Picornaviridae
human cosavirus E1	Picornaviridae
astrovirus VA1	Astroviridae
human papilloma virus 116	Papillomaviridae
klassevirus	Picornaviridae
Lujo virus	Arenaviridae

### (f) New virus families

The discovery curve for virus families is shown in figure 1*b*. Here, a family is included on the date of the first published report of human infection by a virus species from that family. There is too little data (*n* = 23) for detailed statistical analysis, but the figure does suggest a possible decrease in the rate of discovery, implying that the pool of undiscovered families may be relatively modest (see [5]).

Strikingly, no new families have been added to the list since 1988, the longest such interval on record. However, several viruses (specifically Torque Teno (TT) virus, TT mini virus and TT midi virus) newly reported since 1988 remain unassigned to a family.

It should also be noted that there are three virus families that, although they do not contain any known human virus species, do contain species that infect other mammals: Arteriviridae (several species including simian haemorrhagic fever virus); Asfarviridae (African swine fever virus); Circoviridae (including mammal infecting circoviruses as well as gyrovirus which infects chickens). This suggests that the list of families containing human viruses may not yet be complete.

## 3. Emergence as a biological process

### (a) Non-human reservoirs

More than two-thirds of human virus species are zoonotic, i.e. they are capable of infecting vertebrate hosts other than *Homo sapiens* (disregarding invertebrate vectors) [25,26]. By far the most important non-human host taxa are other mammals, with rodents and ungulates most commonly identified as alternative hosts, followed by primates, carnivores and bats. A minority of the zoonotic viruses (less than 20%) are also known to infect birds; very few have been reported from vertebrates other than mammals or birds.

The remaining viruses, as far as we are aware, only naturally infect humans (these are sometimes referred to as ‘specialist’ human pathogens [27]). Some of these (e.g. hepatitis B) may have co-evolved with humans over very long time periods [28]; others (e.g. HIV-1) have much more recent origins [29]. Some of both kinds are believed to have originated in other mammal or bird species [30], including: HIV-1 (derived from a simian immunodeficiency virus found in chimpanzees); HIV-2 (sooty mangabeys); severe acute respiratory syndrome virus (SARS; horseshoe bats); hepatitis B, human T-lymphotropic virus (HTLV)-1 and -2, dengue and yellow fever (all primates); human coronavirus OC43, measles, mumps and smallpox (all livestock); and influenza A (wildfowl). However, we do not know the origins of the majority of specialist human viruses, a gap in knowledge that has prompted calls for an ‘origins initiative’ [30].

### (b) Pathogen pyramid

**Figure 3.**
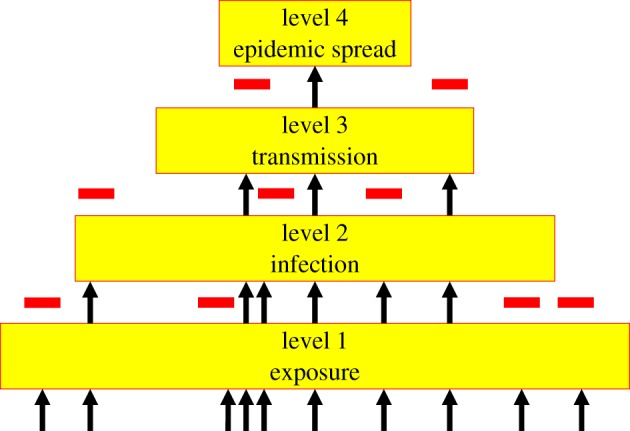
The pathogen pyramid (adapted from [30]). Each level represents a different degree of interaction between pathogens and humans, ranging from exposure through to epidemic spread. Some pathogens are able to progress from one level to the next (arrows); others are prevented from doing so by biological or ecological barriers (bars)—see main text.

A useful conceptual framework for thinking about the emergence of novel viruses is the pathogen pyramid [30,31] (figure 3). The pyramid has four levels.

Level 1 represents the exposure of humans to a novel pathogen; here, a virus. The source of viruses of interest is most likely to be other mammals or birds (see above) and ‘exposure’ implies any route by which a particular viral infection might be acquired, whether by contact with blood, saliva or faeces, contamination of food and water or via an arthropod vector. The rate of such exposure is determined by a combination of the distribution and ecology of the non-human host and human activities. It is likely that exposure to non-human viruses occurs commonly: a process referred to as ‘chatter’ [32].

Level 2 represents the subset of viruses that are capable of infecting humans—that is, overcoming the ‘species barrier’. This is likely to reflect both the molecular biology of the virus (e.g. is it capable of entering and replicating in human cells?—see §3*e*) and the physiology of the exposed human (especially immunocompetence).

Level 3 represents the subset of viruses that can not only infect humans but can also be transmitted from one human to another (by whatever route, including via arthropod vectors). Again, this will mainly reflect the host–pathogen interaction, especially whether it is possible for the virus to access tissues from which it can exit the host, such as the upper respiratory tract, lower gut, urogenital tract, skin or (for some transmission routes) blood.

Level 4 represents the subset of viruses that are sufficiently transmissible between humans to cause major outbreaks and/or become endemic in human populations without the requirement of a non-human reservoir. This equates with the epidemiological condition *R*_0_ > 1 [33], i.e. a single primary case generates, on average, more than one secondary case. This is a function of both the transmissibility of the virus (how infectious an infected host is, and for how long) and properties of the human population (how human demography and behaviour affect opportunities for transmission).

From previous reviews of the literature [25,26,34], it is possible to put approximate numbers of virus species at each level of the pyramid. We know that there are greater than 200 viruses at least at level 2 (see §2*a*). We do not have a good estimate of the total species diversity of mammalian and avian viruses; however, we can get an indirect indication of the magnitude of the barrier between level 1 and level 2. It has been reported elsewhere (R. Critchlow 2010, personal communication) that of the virus species known to infect domestic animals (livestock and companion animals)—to which humans are presumably routinely exposed—roughly one-third are also capable of infecting humans. The species barrier exists: but it is clearly very leaky. Based on data from [25], roughly 50 per cent of the viruses that can infect humans can also be transmitted by humans (level 3), and roughly 50 per cent of those are sufficiently transmissible that *R*_0_ may exceed one (level 4). That a significant minority of (mammalian or avian) viruses should be capable of extensive spread within human populations (or of rapidly becoming so [35]) is consistent with experience: there are several examples within the past hundred years alone (HIV-1, SARS, plus variants of influenza A) and many more in the past few millennia (e.g. measles, mumps, rubella, smallpox). It is noteworthy that the ‘shape’ of the pathogen pyramid for viruses is very different to that for other kinds of pathogen (bacteria, fungi, protozoa or helminths), of which much smaller fractions are capable of extensive spread in human populations (data from [25]). The most straightforward explanation for this is the much more rapid evolution of viruses (especially RNA viruses), allowing them to adapt to a new (human) host much more quickly than other kinds of pathogen.

### (c) Drivers of emergence

Several reviews [10,26,36] have listed so-called ‘drivers’ of the emergence of novel viruses or other pathogens. These constitute a diverse set of environmental and biological factors, many of which—such as ‘urbanization’ or ‘land use’—seem intuitively reasonable but are too broad to relate to mechanistic causes of emergence. Moreover, identification of drivers is usually a subjective exercise: there are very few formal tests of the idea that a specific driver is associated with the emergence of a specific pathogen or set of pathogens. In many cases, this would be a challenging exercise: many drivers have only indirect effects on emergence (e.g. climate change, which is often linked with changing distributions of disease vectors); and often an emergence event has multiple causes (good examples would be the emergence of Nipah virus or SARS coronavirus).

Other ideas about drivers of emergence are even harder to test formally. One such is that we are currently living through a ‘perfect storm’ in which many potential drivers of emergence events (such as population growth, urbanization, global travel and trade, intensification of livestock production) are acting in concert (L. King 2005, personal communication). Upward trends in many drivers can be quantified, but it is not entirely clear that the frequency of emergence events is increasing: one recent study suggested that it increased during the first decade of the HIV/AIDS pandemic, but has decreased thereafter [9].

A slightly different way of thinking about drivers of emergence is to draw an analogy between emerging pathogens and weeds (A. Dobson 2002, personal communication). The idea here is that there is a sufficient diversity of pathogens available—each with their own biology and epidemiology—that *any* change in the human environment (but especially in the way that humans interact with other animals, domestic or wild) is likely to favour one pathogen or another, which responds by invading the newly accessible habitat. This idea would imply that emerging pathogens possess different life-history characteristics to established, long-term endemic pathogens. As noted earlier, the most striking difference identified so far is that the majority of recently emerging pathogens are viruses rather than bacteria, fungi, protozoa or helminths.

### (d) Species jumps

For viruses, one of the key steps in the emergence process is the jump between one host species and humans [37]. (For other kinds of pathogen, there may be other sources of human exposure, notably environmental sources or the normally commensal skin or gut flora). Various factors have been examined in terms of their relationship with a pathogen's ability to jump into a new host species; these include taxonomic relatedness of the hosts, geographical overlap and host range.

Two recent studies provide good illustrations of the roles of host relatedness and geographical proximity. Streicker *et al*. [38] found associations between the degree of cross-species transmission of bat lyssaviruses and both the geographical overlap between bat populations across the USA and the phylogenetically relatedness of the bat species involved. Davies & Pedersen [39] found that primate species tended to share more parasite species if they were both more closely related and had sympatric distributions.

A broad host range is also associated with the likelihood of a pathogen emerging or re-emerging in human populations [26]. An illustrative case study is bovine spongiform encephalopathy (BSE). After BSE's emergence in the 1980s, well before it was found to infect humans (as vCJD), it rapidly became apparent that it could infect a wide range of hosts, including carnivores. This was in marked contrast to a much more familiar prion disease, scrapie, which was naturally restricted to sheep and goats. With hindsight, this observation might have led to public health concerns about BSE being raised earlier than they were.

Host range is a highly variable trait among viruses: some, such as rabies, can infect a very wide range of mammals; others, such as mumps, specialize on a single species (humans). Moreover, for pathogens generally, host range seems to be phylogenetically labile, with even closely related species having very different host ranges [27]. Clearly, the biological basis of host range is relevant to understanding pathogen emergence.

### (e) Cell receptor usage and host range

One likely biological determinant of the ability of a virus to jump between species is whether or not they use a cell receptor that is highly conserved across different (mammalian) hosts. We therefore predicted that viruses that use conserved receptors ought to be more likely to have a broad host range.

To test this idea, we first carried out a comprehensive review of the peer reviewed literature and identified 88 human virus species for which at least one cell receptor has been identified. Although this is only 40 per cent of the species of interest, 21 (of 23) families were represented; so this set contains a good cross-section of relevant taxonomic diversity. Of these 88 species, 22 use non-protein receptors (e.g. heparin sulphate) and, of the remainder, two of the proteins were not entered in the UniProt database [40] (making it impossible to determine whether the protein was ‘conserved’ or not—see below for details), leaving 64 species from 16 families.

On the basis of a previously published study of virus host ranges [26], we accorded these viruses either a ‘narrow’ host range (if the only non-human hosts they were known to infect were other primates) or a ‘broad’ host range (if they were known to infect also other kinds mammals or birds). Using the UniProt database, we determined whether the cell receptor protein was ‘conserved’ by quantifying the amino acid sequence homology between humans and mice. (For the subset of proteins where amino acid sequences data were also available for cows, pigs or dogs, we found very similar patterns.)

**Figure 4.**
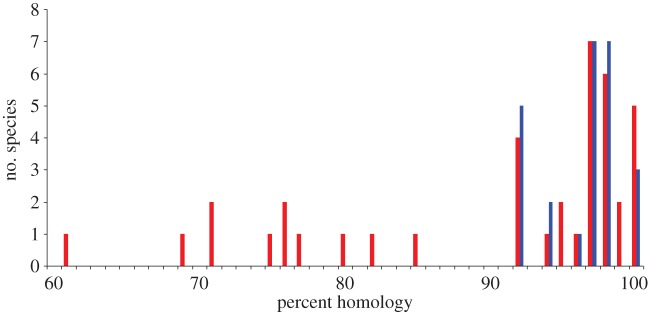
Number of virus species with broad (blue bars) or narrow (red bars) host range as a function of the percent homology of the cell receptor used (see main text).

The result is shown in figure 4. The most striking feature of the plot is that there are no examples of human viruses with broad host ranges that do not use highly conserved cell receptors (i.e. more than 90% amino acid sequence homology). Statistical analyses requires correction for phylogenetic correlation: viruses in the same family are both more likely to use the same cell receptor and more likely to have a narrow or broad host range. This can be crudely (but conservatively) allowed for by testing for an association between host range and receptor homology at the family, not species, level. This gives a statistically significant result (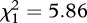, *p* = 0.015).

We conclude that the use of a conserved receptor is a necessary but not sufficient condition for a virus to have a broad host range encompassing different mammalian orders. It follows that a useful piece of knowledge about a novel mammalian virus, helping to predict whether or not it poses a risk to humans, would be to identify the cell receptor it uses. However, this may not always be practicable: at present, we do not know the cell receptor used by over half the viruses that infect humans, and this fraction is considerably smaller for those that infect other mammals.

## 4. Conclusions

The lines of evidence described earlier combine to suggest the following tentative model of the emergence process for novel human viruses. First, humans are constantly exposed to a huge diversity of viruses, though those of others mammals (and perhaps birds) are of greatest importance. Moreover, these viruses are very genetically diverse and new genotypes, strains and species evolve rapidly (over periods of years or decades). A fraction of these viruses (both existing and newly evolved) are capable of infecting humans. It is not clear whether some of these human-infective viruses will already be capable of reaching higher levels of the pathogen pyramid—so-called ‘off-the-shelf’ pathogens—or whether subsequent evolution of their ability to infect and transmit from humans is usually required—‘tailor-made’ [31]. The distinction is potentially important as it implies different determinants of the rate of emergence of viruses with epidemic or pandemic potential: for off-the-shelf pathogens this rate is largely driven by the rate of human contact with a diversity of virus genotypes (possibly rare genotypes) within the non-human reservoir (i.e. ecology); for tailor-made viruses, the key variable is likely to be the rate of genetic adaptation within the new human host (i.e. evolution) [35].

Whichever of these two models is correct (perhaps both), there is a clear implication that the emergence of new human viruses is a long-standing and ongoing biological process. Whether this process will eventually slow down or stop (if the bulk of new virus species constitute extant diversity) or whether it will continue indefinitely (if a significant proportion of newly discovered virus species are newly evolved) remains unclear, although this makes little difference to immediate expectations. There is a hint, from the slower accumulation of new virus families found in humans, that virus diversity may be bounded, but that does not preclude there being a much larger number of virus species ‘out there’ than we are currently aware of. If anthropogenic drivers of this process are important then it is possible that we are in the midst of a period of particularly rapid virus emergence and, in any case, with the advent of new virus detection technologies, we are very likely to be entering a period of accelerated virus discovery. The unavoidable conclusion is that we must anticipate the emergence and/or discovery of more new human viruses in the coming years and decades. By no means all of these will pose a serious risk to public health but, if the recent past is a reliable guide to the immediate future, it is very likely that some will.

The first line of defence against emerging viruses is effective surveillance. This topic has been widely discussed in recent years [10,41], but we will re-iterate a few key points here. Firstly, emerging viruses are everyone's problem: the ease with which viruses can disperse, potentially worldwide within days, coupled with the very wide geographical distribution of emergence events [9], means that a coordinated, global surveillance network is essential if we are to ensure rapid detection of novel viruses. This immediately highlights the enormous national and regional differences in detection capacity, with the vast majority of suitable facilities located in Europe or North America. Secondly, reporting of unusual disease events is patchy, even once detected, reflecting both governance issues and lack of incentives [10]. Thirdly, we need to consider extending the surveillance effort to other mammal populations as well as humans, because these are the most likely source of new human viruses.

Improving the situation will require both political will and considerable investment in infrastructure, human capacity and new tools [10,41]. However, the benefits are potentially enormous. It is possible to forestall an emerging disease event, as experience with SARS has shown. However, our ability to achieve this is closely linked to our ability to detect such an event, and deliver effective interventions, as rapidly as possible. A better understanding of the emergence of new human viruses as a biological and ecological process will allow us to refine our currently very crude notions of the kinds of pathogens, or the kinds of circumstances, we should be most concerned about, and so direct our efforts at detection and prevention more efficiently.
